# Risk estimates for children and pregnant women exposed to mercury-contaminated *Oreochromis niloticus* and *Lates niloticus* in Lake Albert Uganda

**DOI:** 10.1080/23311932.2016.1228732

**Published:** 2016-09-08

**Authors:** Tamale Andrew, Ejobi Francis, Muyanja Charles, Naigaga Irene, Nakavuma Jesca, Micheal Ocaido, Kato Drago, Sente Celsus, Amulen Deborah, Wilson Rumbeiha

**Affiliations:** ^a^ College of Veterinary Medicine, Animal Resources and Biosecurity, Makerere University, P.O. Box 7062, Kampala, Uganda; ^b^ Department of Food Technology and Nutrition, Makerere University, P.O. Box 7062, Kampala, Uganda; ^c^ Veterinary Diagnostic and Production Animal Medicine, College of Veterinary Medicine, Iowa State University of Science and Technology, Iowa, IA, USA; ^d^ Middle East Technical University, Turkey

**Keywords:** mercury, vulnerable populations, fish contamination, risk analysis, disability adjusted life years

## Abstract

Exposure to mercury contaminated fish predisposes populations particularly children and pregnant women to various health hazards including neurotoxicity, reproductive abnormalities and cognitive disorders. Earlier studies in the Lake Albert community have demonstrated the presence of mercury in Nile tilapia and Nile perch. However, the risk estimates for vulnerable groups such as Children and pregnant women is not well documented. Secondary data-set from previous studies were employed comprising family household size and fish consumption history, fish consumption quantity and frequency and mercury levels in fish species in comparison with FAO/WHO guidelines. Data collected was used to establish the hazard quotients (HQs) for the vulnerable group and the general population. A risk model was developed using iRISK to demonstrate the Disability Adjusted Life Years (DALYs) for eating different parts of the fish (muscle and bellyfat). HQ values (HQ = 2.05) above one for the vulnerable group were realized especially with Nile perch muscle. The highest DALYs (0.111) was obtained with tilapia muscle consumption. The study outcome reveals that vulnerable populations are at risk of non-carcinogenic complications. Therefore, there is a need for sensitization of the community especially the vulnerable groups about risks associated with consuming mercury-contaminated fish.

## 1. Introduction

Globally, mercury consumption through fish uptake is considered one of most common routes of exposure for humans (Teisl, Fromberg, Smith, Boyle, & Engelberth, 2011). A primary concern of mercury levels in food is the fact that no amount in foods is considered not to have an ill effect on the population (Johnston & Snow, 2007). The issue of mercury amounts in fish has received considerable critical attention due to the immune, neural, reproductive disorders in adults and in children as well as cognition and mental disorders (Cheng et al., 2013; Teisl et al., 2011).

Several studies have documented the human health risk attributed to mercury exposure in the fish parts of the predominant fish species consumed (Sidhu, 2003; Zeilmaker et al., 2013). This mercury exposure is based on the provisional tolerable weekly intake (PTWI) amounts in fish as per the WHO/FAO guideline of 1.6 μg/kg body weight (Carvalho, Matos, Mateus, Santos, & Batoréu, 2008). Consumption of fish with mercury levels beyond the FAO/WHO guideline shall result into health complications especially in the vulnerable groups of children under 17 years, expectant mothers, and communities who depend on fish for subsistence i.e. fishing communities (Carvalho et al., 2008; Dewailly et al., 2012).

Previous studies reporting risk assessment of mercury have examined the sources, location, population at risk, fish species and their state, and the methods of assessment of the risks. Traditionally, it has been argued that the common sources of mercury are historical sites under industrialization, sites with oil activities, plants i.e. Chloralkali, sediment, water and fish from both natural and anthropogenic sources (Abdallah & Abd-Allah, 2012; Agusa et al., 2005; Al Sayegh Petkovšek, Mazej Grudnik, & Pokorny, 2012; Bravo et al., 2010; Dahshan, Abd-Elall, & Megahed, 2013). To date, several studies have linked mercury exposures to locations (where the vulnerable communities live) especially coastal areas, gold mines, along contaminated rivers and lakes, fishing communities, wetlands, and upstream and downstream sites (Agusa et al., 2007; Bidone, Castilhos, Santos, Souza, & Lacerda, 1997; Bravo et al., 2010; Mansilla-Rivera & Rodríguez-Sierra, 2011; Mansouri, Khorasani, Monavari, Karbasi, & Panahandeh, 2013; Usha & Reddy, 2013; Weldemariam, 2012).

Recent evidence suggest that, the most vulnerable groups to Mercury toxicity are pregnant women, children less than 17 years and women of child bearing age (Arakawa, Yoshinaga, Okamura, Nakai, & Satoh, 2006; Klein, 2005; Mansouri et al., 2013; Tang, Kwong, Chung, Ho, & Xiao, 2009).

Data from several sources have shown that the common fish eaten (in the Lake Albert it is tilapia and Nile perch) and fish parts are associated with different levels of mercury and that the commonly analyzed parts are the muscle, liver, gills, kidney, brain and blood (Andrew, Francis, Charles, Naigaga, Jessica*,* et al., 2016; Mieiro, Pacheco, Pereira, & Duarte, 2009; Sary & Mohammadi, 2011).

To date several studies have used the levels of Mercury in the different fish species and fish parts to estimate the risk for humans and involves hazard ratios, acceptable risk levels, exposure levels, PTWI and transferable factor (Burger & Gochfeld, 2005; Sidhu, 2003; Tang et al., 2009; Zhu, Yan, Wang, & Pan, 2012). One of the shortfalls of these studies is the generalization of the risk across age groups and failure to extrapolate the risk to the Disability Adjusted Life Years (DALYs) accrued; a gap the current work is going to fill for the fishing communities in the Lake Albert.

One of the obstacles to reaping the benefits associated with fish consumption in the Lake Albert community was lack of information on the mercury levels in the predominant fish consumed in fishing communities (Gimou et al., 2013; Guevel, Sirot, Volatier, & Leblanc, 2008; Raissy & Ansari, 2014)**.** This gap was addressed by Andrew, Francis, Charles, Naigaga, Jessica, et al. (2016) but the risk for the vulnerable groups i.e. children less than 17 years and pregnant women were not addressed.

To date, there has been no reliable evidence that mercury uptake through fish bellyfat consumption in the fishing community poses a risk to the vulnerable groups. This study therefore, sheds new light on the DALYs and the hazard quotient (HQ) of mercury for the vulnerable groups i.e. children less than 17 years and expectant mothers in the fishing community (Chan & Jacobs, 2013). This view point about mercury risk for vulnerable group is in agreement with Jiang et al. (2010) who studied expectant mothers in Taiwan and came to the conclusion that fish consumption and daily uptake amounts were the two key factors responsible for Mercury toxicity for the *in vivo* and expectant mothers.

Therefore, this paper argues that without knowledge of the risk posed by mercury levels in fish and fish parts consumed by the vulnerable populations, there is a possibility of HQs greater than one hence non-carcinogenic health complications and/or DALYS.

## 2. Methods

### 2.1. Focus area

The Lake Albert community is located in Hoima district, Uganda. It is one of the most populated with a total population of 535,000 persons (Uganda Bureau of Statistics, 2014). This population shall increase due to oil activities, charcoal burning and access to the fishing sites. The annual population growth rate of the district according to the 2014 National census report was 10.7 per year, the highest in the country. The district has four sub-counties (Buseruka, Kigorobya, Kabwoya, Kyangwali) with landing sites, and these have total human population of 43,018, 68,000, 63,118 and 97,366 persons respectively. The maps below show the location of Hoima and landing sites.


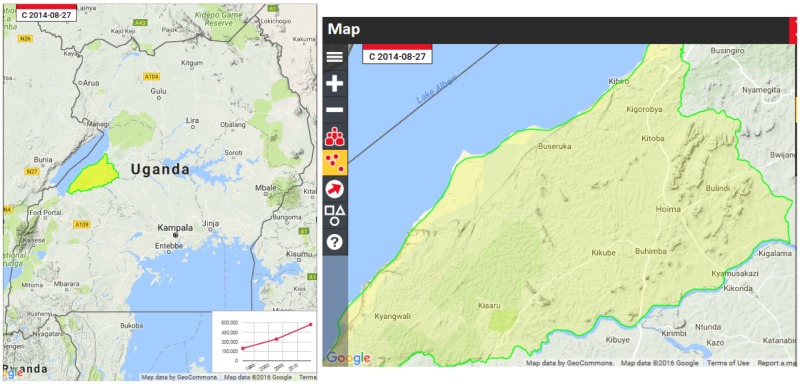
Source: Google Map (Google, 2016).

### 2.2. Data collection

Secondary data was collected from two studies executed in the Lake Albert region. The first study contributed sociocultural information on the household size, frequency of fish consumption, amount of fish consumed, fish species (*Oreochromis niloticus* and *Lates niloticus*) and parts eaten (Andrew, Francis, Charles, Naigaga, Jesca*,* et al., 2016). The second study by (Andrew, Francis, Charles, Naigaga, Jesca, et al., 2016) contributed the mercury amounts in different fish species and parts, the amounts of fish used to generate the mercury quantities and the percentages of the samples above the FAO/WHO guideline values. The secondary data utilized in the analysis are displayed in Table 1.

**Table 1.** Sociocultural factors and fish parameters in Lake Albert

Attribute	Quantity
Median household family size	4
Median total amounts of fish consumed per week (kg)	8
Median mercury concentration Nile perch muscle (mg/kg)	0.0243
Median mercury concentration Nile perch bellyfat (mg/kg)	0.0197
Median mercury concentration tilapia muscle (mg/kg)	0.0179
Median mercury concentration tilapia bellyfat (mg/kg)	0.0157
Percentage of Nile perch muscle above WHO guideline (0.05 mg/kg)	15.15
Percentage of Nile perch bellyfat above WHO guideline (0.05 mg/kg)	21.9
Percentage of tilapia muscle above WHO guideline (0.05 mg/kg)	31.03
Percentage of tilapia bellyfat above WHO guideline (0.05 mg/kg)	4
Total mass of Nile perch muscle (g)	44.5
Total mass of tilapia muscle (g)	51.39
Total mass of Nile perch bellyfat (g)	52.28
Total mass of tilapia bellyfat (g)	46.06

Source : Andrew, Francis, Charles, Naigaga, Jessica, et al. (2016).

The Wilcoxon test was utilized to compare the amounts of mercury found in muscle and bellyfat to that of FAO/WHO guidelines.

Boxes 1 and 2 provided secondary data for imputation in the iRISK model to generate the DALYs from the four scenario of fish consumption (tilapia muscle and bellyfat; Nile perch muscle and bellyfat).

**Box 1: Oral reference doses for mercury non cancer complications**

**Table:** 

	Oral RfD (mg/kg per day)	Source
Children <17 years and women of childbearing age	1 × 10^−4^	Oehha.ca.gov 2008
Men and women above childbearing age	3 × 10^−4^	Oehha.ca.gov 2008

Source: Klasing and Brodberg (2008).

**Box 2: Information used to generate the scenarios for mercury in different fish parts**

**Table:** 

Attribute	Description
Hazard	Mercury; chemical
Food	Fish species (Nile perch/tilapia) and fish part (bellyfat or muscle)
Process model	Mercury in part of the fish species selected
Consumption model	Fish species part consumed
Metric	DALY
Exposure type	Chronic
Converged	Yes (9,000 samples)
Population groups	Children less 5 years; Children between 5–12 years; Teenagers 13–17 years; general population above 18 years and pregnant women
Total span years	105
Dose response	Linear by slope; Slope 0.0001; Probability of adverse effects 100%
DALY/Case	2.88

### 2.3. Data analysis

The two risk values computed from the secondary data was the HQ values for the different fish species and parts and the DALYs accrued as a result.

Before the HQs analysis, the Food chemical dietary exposure was computed as documented by (Carvalho et al., 2008). This was later followed by HQs analysis as documented by (Castilhos et al., 2006). The results are displayed in Table 2.

**Table 2.** Mercury exposures due to fish consumption in fishing community

Exposure for different populations	Nile perch muscle	Nile perch bellyfat	Tilapia muscle	Tilapia bellyfat
Mercury levels in different fish parts (mg/kg)	0.0243	0.0197	0.0179	0.0157
Amounts consumed (kg per day)	2	2	2	2
Frequency of fish consumption (per week)	4	4	4	4
Assumed body weight for four family members’ (kg)	240	240	240	240
Fish mercury dietary exposure (mg/kg per day)	0.0002	0.00016	0.00015	0.00013
HQ for children <17 years and childbearing age women	2.025	1.64167	1.49167	1.30833
HQ for men and women above childbearing age	0.675	0.54722	0.49722	0.43611

In addition, the study uses iRISK software to compute the DALYS encountered by the vulnerable population with exposure levels of mercury in fish as documented by Poulin, Gibb, and Pruss-Ustun (2012).

### 2.4. Limitations

The major limitation to this study is in computation of the DALYs where person categories overlap i.e. pregnant women and women of child bearing age. This overlap is expressed in the life span of 105 years.

## 3. Results

### 3.1. Health risks from mercury exposure through predominant fish consumed

The first set of analyses were targeted towards establishing the HQs of mercury in the Nile perch and tilapia fish parts consumed. What is interesting about the analyses is that the HQs for the vulnerable populations i.e. children less than 17 years and women of childbearing age based on fish part eaten are above one indicative of non-cancer health risks while those of the general population do not show any health risk. The predominantly consumed fish species posed health risks for the children less than 17 years, childbearing mothers and the general population as shown in Table 2:

### 3.2. DALYS attributed to mercury consumption through fish consumption

The loss in production as a result of consuming fish contaminated with mercury can be expressed as DALYs. These DALYs are computed from the life course duration, which for the model had a total of 105 years; total illness which results from amounts of mercury in fish and eating occasions. From the model, it is apparent that the most of the DALYs are accrued from consuming tilapia muscle as compared to other fish parts. Interestingly, the total DALYs for consumption of Nile perch are below that of the tilapia part consumed except tilapia bellyfat. Table 3 shows the DALYs from the four possible scenarios of eating fish in the Lake Albert. Table 3 also shows that the total illness is highest amongst persons who eat tilapia muscle and that the eating occasions are the same for the community.

**Table 3.** Scenario analysis of Mercury related illness through fish consumption

Scenarios	Life course duration	Eating occasions	Total illness	Means of illness	Total DALYs (per year)
Mercury in tilapia (mm)	105	2.72*E* + 5	0.0387000	1.42*E*–7	0.111
Mercury in Nile perch bellyfat	105	2.72*E* + 5	0.0000286	1.05*E–10*	0.0000824
Mercury in Nile perch muscle	105	2.72*E* + 5	0.0000180	6.61*E*–11	0.0000517
Mercury in tilapia bellyfat	105	2.72*E* + 5	3.93*E*-6	1.45*E*–11	0.0000113

## 4. Discussion

The present study was designed to determine the estimated health risks in vulnerable populations due to mercury exposure through fish consumed in Lake Albert. Earlier research in the Lake Albert community exhibited median amounts of mercury in predominant fish species consumed in the Lake Albert community which were far below the FAO/WHO guideline values. This viewpoint, however, contrasts with Johnston and Snow (2007), who observed that all levels of mercury can affect different vulnerable population especially the childbearing women and children less than 17 years. Therefore, there is a need for consideration of the fish species and parts, frequency of consumption and amount consumed on a daily or weekly basis, in order to determine the health risks one is exposed to (Lee et al., 2006). Unless the mercury levels in the fish studied are higher than the normal ranges for guideline values, most studies have stopped at this level and made recommendations to the communities. However, the use of guideline values and their relationship to amounts of Mercury in fish is valid for situations where the level of toxicity in fish muscle is high i.e. in the Khuzestan study by Sary and others who found reported Mercury levels in freshwater fish greater than the 0.05 mg/kg (Sary & Mohammadi, 2011). Therefore, for the case of the vulnerable Albert community with few or no studies, there is a need to utilize the available information to establish the health risks from the fish consumed regardless of the guideline values.

The exposure diet intake is linked to the HQ which signifies the relationship between the exposure obtained in the diet and the oral reference dose for mercury (Castilhos et al., 2006). Choice of the oral reference dose is critical in determining the health risks the vulnerable community is exposed to i.e. use of the general population or vulnerable population oral reference dose (Zhu et al., 2012). The results of this study reveal health risk when HQs were computed for the vulnerable population in the community. The HQ values were higher than one for all parts of the fish consumed and Nile perch muscle had a HQ value of two. These results further support the observations Tang et al. (2009) made about mercury levels in fish consumed by school going children and was able to predict that frequency of consumption and amounts which will predispose the community to toxic levels of Mercury Tang et al. (2009). Although the amounts of Mercury in fish parts were little in the fish, the frequency of consumption exposed the children less than 17 years and women of childbearing age to non-carcinogenic risks. Therefore, there is a need to send out a message for this vulnerable group about the health hazard they are encountering daily by consumption of the fish. Use of specific messages for different target groups was demonstrated in the USA during a study by Klein (2005) which involved pregnant women and children and observed that there is a need for a unique message for the vulnerable group.

Hazard Index (HI) for both vulnerable and general populations if computed for the fish parts consumed spells out the health risks. For the vulnerable populations, if the HQs from the study are added, then the HI is greater than one and the same holds for the general populations. An HI greater than one spells out probable health risks from the mercury consumed. These results are in agreement with Poulin, Gibb, and Pruss-Ustun (2012) who documented higher HI levels in carnivorous than herbivorous fish a pointer towards the HI points towards non-carcinogenic risk attributed to mercury uptake in fish parts especially the Nile perch.

However, the findings of the current study do not support the previous research with other authors who examined predominant fish species consumed by the natives of the Amazon and found that fish species whether omnivorous or carnivorous did not bioaccumulate mercury differently in the tissues studied (Bidone et al., 1997). A possible explanation for this may be that the source of contamination and its proximity to the nearby lakes or rivers. For the Lake Albert community, the primary source was oil exploration followed by natural activities, and some oil wells were close to the landing sites. One unanticipated finding was that the bellyfat accumulated less mercury than the muscle. This fact is useful as part consumed by vulnerable populations in areas where the health risk due to levels of Mercury is high, especially in the muscles. These results, however, need to be interpreted with caution since no guideline values from FAO/WHO is documented for bellyfat except this study.

On the question of mercury burden, the study found out that the highest burden was through consumption of muscle from tilapia. This burden was in contrast to what had been earlier predicted where carnivorous risk show the highest burden of disease (Poulin et al., 2012). This inconsistency may be due to access to fish, and amounts consumed. Since more people access tilapia than Nile perch in the market in the Lake Albert community and district, more DALYs are attributed to its consumption. Therefore, empirical studies need to be carried out before messages are sent out on which species to consume and the associated health risks. Unlike in Canada where vulnerable populations are limited to less than three meals of fish per week to avoid the risks attributed to mercury, the Lake Albert community has no such limitation since the eating occasions established to cause strife were way beyond 700 meals per day (Hursky & Pietrock, 2012). The other alternative to determine the burden in humans would be the use of levels of mercury in hair. This concept, however, has shown inconsistency with the amounts attributed to fish exposure especially for pregnant women (Arakawa et al., 2006). These findings have important health implications for developing country fishing communities.

## 5. Conclusions and recommendations

Presence of HQs above one signify a non-carcinogenic risk for the vulnerable groups in the Lake Albert fishing community. The highest DALYs for the vulnerable group occurred with consumption of tilapia muscle. In order to avoid further consequences from the above, there is need for community sensitization about risks associated with mercury especially for the vulnerable group. There is also need to investigate the amount of mercury in blood and hair of the children and pregnant women in the Lake Albert.

## Funding

This research was supported by the Consortium for Advanced Research Training in Africa (CARTA). CARTA is jointly led by the African Population and Health Research Center (APHRC) and the University of the Witwatersrand and funded by the Wellcome Trust (UK) [grant number 087547/Z/08/Z], the Department for International Development (DfID) under the Development Partnerships in Higher Education (DelPHE), the Carnegie Corporation of New York [grant number B 8606], the Ford Foundation [grant number 1100-0399], Google.Org [grant number 191994], Sida [grant number 54100029], MacArthur Foundation [grant number 10-95915-000-INP] and British Council. This work was also supported by IAEA UGA5035.

## Public Interest Statement

Health in developing countries is achievable through sound governance, based on information generated from the field. This situation is replayed in the hard to reach areas and populations where education is limited. The limited education translates into increased exposure to chemicals which are harmful. The picture in the Lake Albert community shows a situation where the common source of protein i.e. Fish turned out to be the source of risk for children and women of childbearing age. If data interpretation stops at the general population, nothing of this magnitude is reported by the research team. Now, it is clear that consumption of Nile perch muscle presented some risk to the children and mothers of childbearing age. Therefore, there is the need for constant checks on what the people are eating to avoid the diseases from chemicals and other contaminants
